# The Relevance of the Virchow Node and Virchow Triad in Renal Cancer Diagnosis

**DOI:** 10.3390/clinpract15010018

**Published:** 2025-01-14

**Authors:** Luiza-Roxana Dorobantu-Lungu, Viviana Dinca, Andrei Gegiu, Dan Spataru, Andreea Toma, Luminita Welt, Mihaela Florentina Badea, Constantin Caruntu, Cristian Scheau, Ilinca Savulescu-Fiedler

**Affiliations:** 1 Department of Cardiology, Emergency Institute for Cardiovascular Diseases “C.C. Iliescu”, 022328 Bucharest, Romania; luiza-roxana.dorobantu@rez.umfcd.ro; 2 Department of Cardiology and Internal Medicine, Colțea Clinical Hospital, 030167 Bucharest, Romania; vivianadinca01@gmail.com (V.D.); andrei-florentin.gegiu@rez.umfcd.ro (A.G.); dvspataru@gmail.com (D.S.); ilinca.savulescu@umfcd.ro (I.S.-F.); 3 Department of Otorhinolaryngology (ORL), Colțea Clinical Hospital, 030167 Bucharest, Romania; andreea.toma@umfcd.ro; 4 Department of Otorhinolaryngology, “Carol Davila” University of Medicine and Pharmacy, 050474 Bucharest, Romania; 5 Department of Pathological Anatomy, Colțea Clinical Hospital, 030167 Bucharest, Romania; luminita.welt@coltea.ro; 6 Department of Radiology and Medical Imaging, Colțea Clinical Hospital, 030167 Bucharest, Romania; mihaelaflorentinabadea@gmail.com; 7 Department of Physiology, “Carol Davila” University of Medicine and Pharmacy, 050474 Bucharest, Romania; 8 Department of Dermatology, “Prof. N.C. Paulescu” National Institute of Diabetes, Nutrition and Metabolic Diseases, 011233 Bucharest, Romania; 9 Department of Radiology and Medical Imaging, “Foisor” Clinical Hospital of Orthopaedics, Traumatology and Osteoarticular TB, 030167 Bucharest, Romania; 10 Department of Internal Medicine, “Carol Davila” University of Medicine and Pharmacy, 050474 Bucharest, Romania

**Keywords:** Xp11.2 translocation, TFE3 gene fusion, Virchow node, clinical diagnosis, medical imaging, multidisciplinary management, differential diagnosis, renal tumor

## Abstract

**Background:** The purpose of this article is to overview the clinical significance of left supraclavicular adenopathy and review the etiology of inferior vena cava (IVC) thrombosis, starting from a presentation of a rare case of renal cell carcinoma (RCCs) with Xp11.2 translocation involving TFE3 gene fusion. This article also aims to review the literature to understand the characteristics of this rare type of renal tumor. Renal cell carcinoma (RCC) associated with Xp11.2 translocation/gene fusion TFE3 is a rare subtype of kidney cancer that was classified in 2016 as belonging to the family of renal carcinomas with MiT gene translocation (microphthalmia-associated transcription factor). The prognosis for these kidney cancers is poorer compared to other types. **Methods:** We present a case of a 66-year-old man with Virchow–Troisier adenopathy during physical examination, which raises the suspicion of infra-diaphragmatic tumor. The echocardiography highlighted a heterogeneous mass in the right cardiac cavities, and the abdominal ultrasound exam revealed a solid mass at the upper pole of the left kidney. **Results:** Following computed tomography, magnetic resonance imaging, PET-CT, and histopathological and immunohistochemical examinations, the patient was diagnosed with renal carcinoma with Xp11.2 translocation and TFE3 gene fusion. **Conclusions:** IVC thrombosis is often associated with neoplastic disease due to the procoagulant state of these patients, the most common malignancies related to IVC thrombosis being represented by RCCs (38%), genitourinary cancers (25%), bronchus and lung cancers, retroperitoneal leiomyosarcoma, and adrenal cortical carcinoma. Imaging methods play a crucial role in differential diagnosis, allowing for the localization of the primary tumor and assessment of its characteristics.

## 1. Introduction

Over 50% of renal cell carcinoma (RCC) cases are diagnosed incidentally due to their long asymptomatic evolution. Over 30% of these patients have metastatic disease at the time of diagnosis [1]. Unfortunately, the classical presentation, represented by macroscopic hematuria, abdominal mass, abdominal pain, and varicocele, is rarely seen. Isolated gross hematuria is the most frequent alarm sign for RCC, occurring in over 60% of patients. Of note, paraneoplastic syndromes occur frequently and are mainly represented by hypercalcemia, erythrocytosis, Cushing syndrome, and increased prolactin [2]. Nearly 20–25% of patients with RCC have vascular thrombosis, and the tumoral thrombus may extend into the IVC up to the right atrium [3]. The median survival rate is about 5 months, and about 29% at 1 year from the diagnosis [4].

RCC (also known as Grawitz tumor) represents the most common malignant tumor of the kidney, the major subclasses of RCC being represented by clear cell RCC, papillary RCC, and chromophobe RCC [5].

Renal carcinoma associated with Xp11.2 translocation/gene fusion TFE3 is a rare subtype of kidney cancer that was recognized as a distinct entity in 2004 and classified in 2016 as belonging to the family of renal carcinomas with MiT gene translocation (Microphthalmia-associated transcription factor). The main features of this type of renal cancer, as stated by its name, are fusions involving the TFE3 gene, situated on chromosome Xp11.2, resulting in the overexpression of the TFE3 protein within the nuclei of cancer cells [6]. The incidence of Xp11.2 translocation RCC is relatively low. Previous studies have reported its incidence as 0.9% of adult RCC cases, 15% of young adult RCC cases, and 54% of child RCC cases. The prognosis for these kidney cancers is poorer compared to other types, especially in the adult population [6].

Several neoplasms can be mistaken for Xp11 translocation renal cell carcinoma, particularly clear cell and papillary renal cell carcinomas. While imaging techniques remain the best methods for staging and monitoring renal carcinomas, histopathological and especially immunohistochemical evaluations are the most reliable for accurate diagnosis in the context of differential diagnosis. While positive staining for the TFE3 gene in immunohistochemical examinations has been a hallmark of renal carcinoma with TFE3 gene fusion, the diagnosis is now confirmed through more precise gene karyotype detection and FISH analysis using paraffin-embedded sections or formalin-fixed tissue [6].

Because of the rarity of this disease in adults, our understanding of its pathogenesis and treatment is incomplete [6]. Currently, an effective treatment for this type of kidney cancer is not known [7].

RCC can metastasize to any location in the body, and distant metastases are common, being present in 30% to 40% of patients with metastatic disease. The most common sites affected are the lungs (76%), regional lymph nodes (LN) (66%), bone (42%), and liver (41%). However, only 1.5 to 3.5% of RCC patients show solitary metastasis, and only 1% have metastasis confined to the head and neck [8].

Supraclavicular lymphadenopathy (Virchow node) is a significant sign of malignancy. When metastasis is detected in the left supraclavicular node in patients, locating the primary cancer remains a difficult and time-consuming challenge despite the dramatic development of screening technologies; therefore, a biopsy of the Virchow node can become essential for the definitive diagnosis [9].

Metastasis from an infra-diaphragmatic tumor to the left supraclavicular lymph node occurs via the rich lymphatic network of the retroperitoneal lymph nodes, cisterna chyli, and thoracic duct, which drains into the systemic circulation via the left subclavian vein. Supradiaphragmatic tumor metastases to the lymph nodes of the head and neck without lung involvement are considered to occur via the vertebral venous plexus system.

Morphologically, the vertebral venous plexus system shows interindividual variability with few or no valves and numerous branches. Therefore, tumor nests encounter little resistance as they ascend through the vertebral venous plexus when the intra-abdominal or intrathoracic pressure is increased, and these tumor nests do not pass through the lung [9].

Several studies and case reports in the literature indicate that when supraclavicular lymphadenopathy is present without a primary tumor, or when a tumor is identified but poses a prohibitive risk for biopsy, diagnosis relies on obtaining tissue and conducting immunohistochemical (IHC) staining of the metastasis [10]. However, documented cases presenting with Virchow sign in renal cancers are rare [8,11,12].

### 1.1. Patient Information

To emphasize the importance of thorough clinical examination in diagnosing conditions, we present a case of a 66-year-old patient from a rural area, admitted to the Department of Cardiology and Internal Medicine, Colțea Clinical Hospital in Bucharest, for physical fatigue, early satiety, and weight loss (approximately 15 kg in the last 2–3 months). It is noteworthy that the patient denied ever having abdominal pain or gross hematuria. He was a non-smoker with no known family history of cancer.

At admission, he was slightly tachycardic with a heart rate of 107 beats per minute, normotensive with a blood pressure of 130/80 mmHg, and afebrile, with a saturation of 97% on room air.

From the personal pathological history, we note the following: arterial hypertension stage 2, type 2 diabetes mellitus under oral antidiabetic treatment, dyslipidemia, and obesity.

### 1.2. Clinical Findings

The physical examination revealed cutaneous–mucosal pallor; a painless, hard, slightly mobile left supraclavicular adenopathy on adjacent planes approximately 3 × 3 cm in size; moderate bilateral leg edema; and sensitivity at palpation in the left hypochondrium, with no other pathological changes.

Laboratory tests showed moderate normochromic, normocytic anemia, an inflammatory syndrome with elevated D-dimers, and azotemia syndrome. Moreover, the urinalysis showed no microscopic hematuria, with proteinuria and the urine culture being negative for common pathogens (Table 1 and Table 2).

### 1.3. Diagnostic Assessment

**Figure 1:**
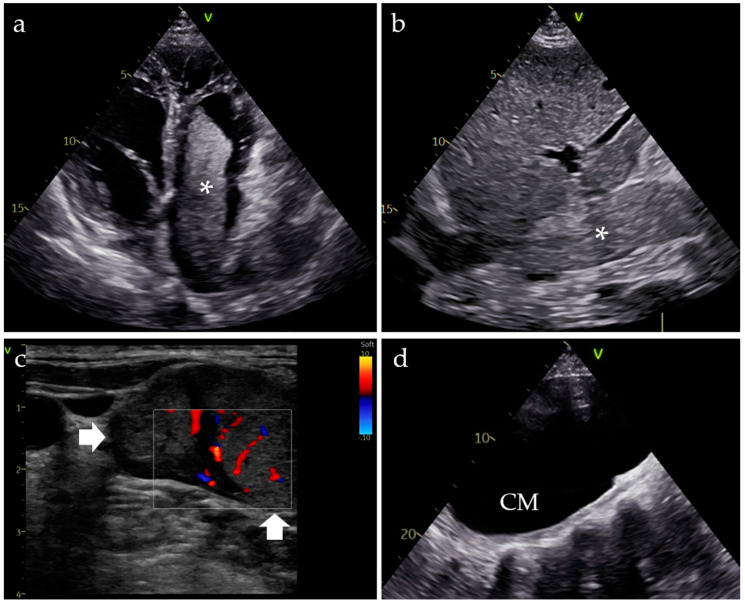
(**a**) Hyperechoic mass protruding from the right atrium into the right ventricle in diastole (*). (**b**) Thrombotic mass occupying almost the entire lumen of the inferior vena cava (*). (**c**) Enlarged lymph node in the supraclavicular fossa, with enhanced Doppler signal (arrows). (**d**) Large cystic mass (CM) in the lower pole of the left kidney.

Transthoracic echocardiography revealed a mobile, hyperechoic mass occupying 80% of the surface of the right atrium, protruding into the right ventricle in the diastole and nearly completely obstructing the inferior vena cava (IVC) lumen. Additionally, there was moderate tricuspid regurgitation and moderate dilation of the right chambers, with a pulmonary artery pressure (PAP) of 35 mmHg. The echocardiographic appearance, highlighting extensive thrombosis from the IVC towards the right ventricle, was highly suggestive of tumoral thrombosis (Figure 1a,b). Cervical ultrasound revealed the supraclavicular mass (Figure 1c), and an abdominal ultrasound was also performed. A transonic mass was detected at the inferior pole of the left kidney, measuring approximately 20 cm (Figure 1d). A heterogeneous, hyperechoic mass, seemingly originating from the upper pole of the left kidney, was also visualized.

The coexistence of Virchow–Troisier adenopathy, a renal cystic mass, and an upper pole mass in the left kidney required further imaging investigations.

An unenhanced CT scan was performed, with no contrast media administration due to renal dysfunction (glomerular filtration rate—GFR 37.36 mL/min/1.73 m^2^). While the lack of contrast media generally limits the amount of data provided, in this case, the diagnostic elements were clearly identified. The CT examination revealed a retroperitoneal, median and paramedian, poly-nodular mass with an irregular, bosselated contour, measuring a maximum of 13.4 × 10 × 12.5 cm. The mass appeared hetero-dense on the unenhanced scan, predominantly showing tissue densities with some areas of fluid–para-fluid densities. It encompassed and obliterated the demarcation interface with the inferior vena cava (IVC) with abdominal aorta involvement.

**Figure 2:**
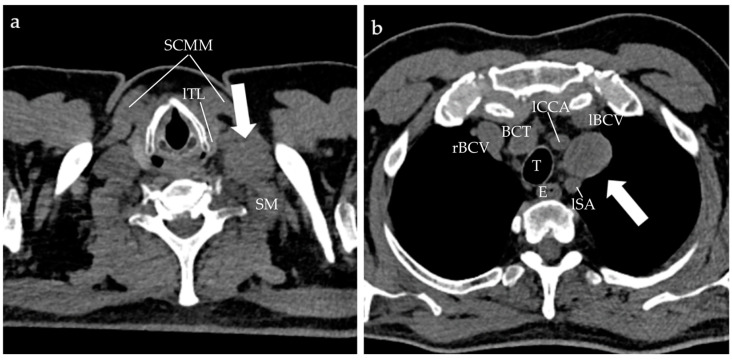
Unenhanced CT scan of the neck (**a**) and thorax (**b**), showing left supraclavicular lymphadenopathy (arrow in (**a**)) and left paratracheal lymphadenopathy (arrow in (**b**)). SCMM = sternocleidomastoidian muscle; SM = scalenus muscle; lTL = left thyroid lobe; rBCV = right brachiocephalic vein; lBCV = left brachiocephalic vein; BCT = brachiocephalic trunk; lCCA = left common carotid artery; lSA = left subclavian artery; T = trachea; E = esophagus.

The described tumor was in direct contact with the left kidney (maintaining the demarcation interface), the inferior duodenal flexure, the third part of the duodenum (DIII), and the right psoas muscle. Cranially to the mass, the IVC measured an increased caliber of up to 4.5 cm. Additionally, a renal cortical cyst on the left side was detected, measuring 12 × 17.6 × 20.3 cm, exerting a mass effect on the left kidney, displacing it anteriorly and above the left psoas muscle. Left supraclavicular lymphadenopathy along with paratracheal adenopathy measuring 3.5 × 2.8 cm and numerous mediastinal and abdominal adenopathies were also visualized (Figure 2).

**Figure 3:**
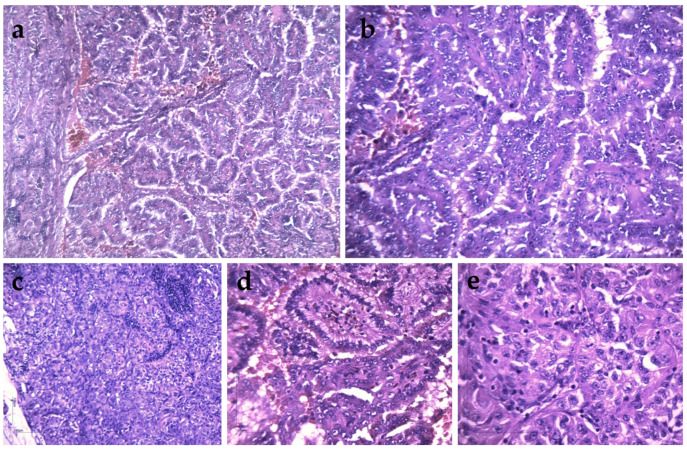
Histopathological examination of Virchow–Troisier adenopathy. (**a**) TFE3 rearranged carcinoma with papillary and solid growth pattern, composed of clear to eosinophilic pseudostratified cells and high-grade nuclei. HE 10×; (**b**) TFE3 rearranged carcinoma with papillary architecture. HE 20×; (**c**) lymph node metastasis. Solid growth pattern of tumor proliferation. HE 20×; (**d**) microscopic detail of papillary structures, composed of cells with eosinophilic and clear cytoplasm and high-grade nuclei. Hemosiderin deposits are also observed. HE 40×; (**e**) microscopic detail of the solid areas, showing cells with predominantly eosinophilic cytoplasm and high-grade nuclei. Relatively frequent mitotic figures are observed. HE 60×.

The excision of left supraclavicular adenopathy was performed, followed by histopathological and immunohistochemical examination. In complex cases such as this one, the interpretation is performed by at least two pathologists in order to minimize interpretation bias; the team reached an agreement and validated the findings. The histopathological analysis staged the lymph node metastasis as renal papillary carcinoma without capsular invasion (Figure 3).

**Figure 4:**
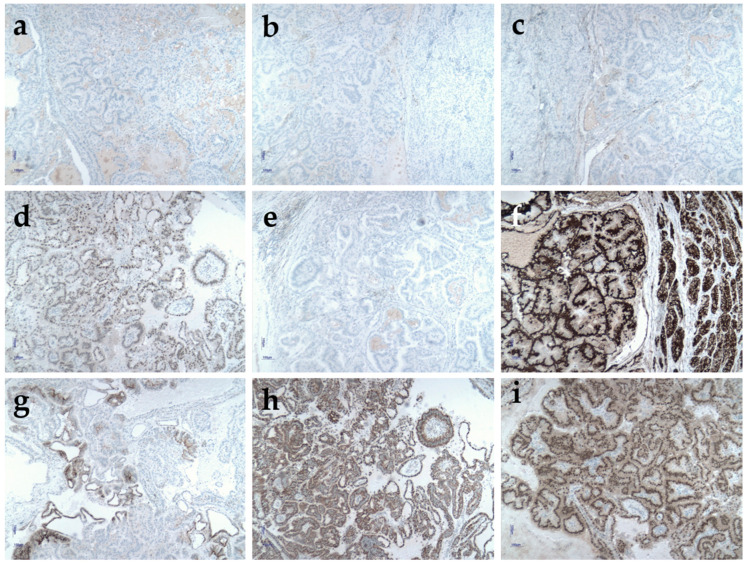
Immunohistochemical examination of Virchow–Troisier adenopathy. (**a**) Negative TTF1; (**b**) negative thyreoglobulin; (**c**) negative CK7; (**d**) diffuse nuclear positivity for TFE3; (**e**) negative ER; (**f**) strong diffuse expression for vimentin; (**g**) focal positivity for CA IX; (**h**) conserved SDHB expression in the tumor cells; (**i**) strong nuclear expression for PAX8.

Immunohistochemically, positive staining was observed for TFE3, Vimentin, CA IX, SDHB, and PAX8, with negative staining for TTF1, Thyroglobulin, CK7, and ER. This pattern is highly suggestive of secondary lymph node involvement of renal carcinoma with Xp11.2 translocation, involving TFE3 gene fusion (Figure 4).

TFE3 is highly sensitive for TFE3-rearranged RCC (positive in 95% of cases), though it may also be overexpressed in other tumor types, such as epithelioid angiomyolipoma, paragangliomas, melanomas, perivascular epithelioid cell tumors, and adrenocortical carcinomas [13,14,15,16,17,18]. However, the histological features in this case exclude these differential diagnoses. Key considerations included papillary RCC and clear cell papillary RCC, both excluded by diffuse TFE3 positivity. Additionally, CA IX would typically show diffuse positivity in clear cell papillary RCC, which was not observed here [19].

Other potential origins, such as epithelial tumors of thyroid, ovarian, endometrial, and pulmonary origins, were excluded based on patient gender, pulmonary CT, and negative staining for TTF1, thyroglobulin, and ER. CK7 negativity was noted, which is occasionally absent in eosinophilic tumors, consistent with this case [20].

The patient has completed additional investigations that could not be technically performed at our hospital at that time, including MRI and PET-CT.

**Figure 5:**
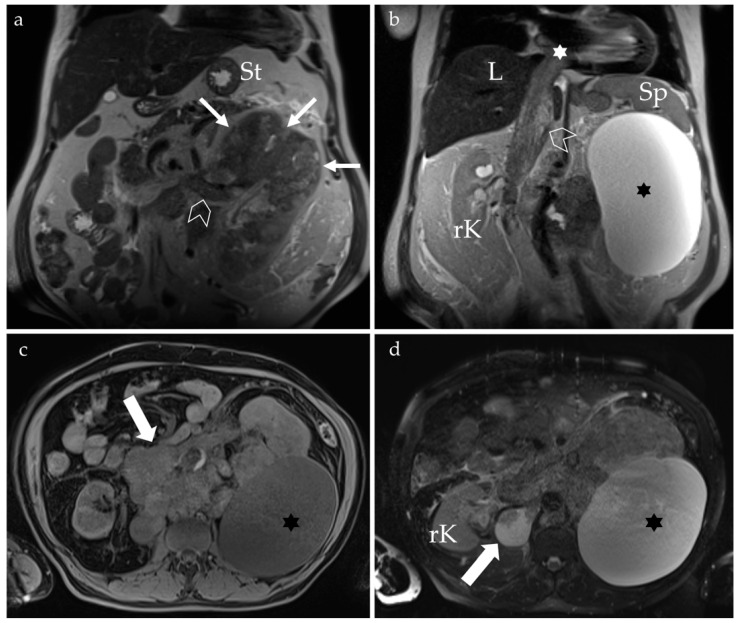
MRI examination of the abdomen, T2 weighted sequence in the coronal plane (**a**,**b**). A large left renal tumor (arrows), with tumor thrombus in the left renal vein (chevron, (**a**)) and inferior vena cava (chevron, (**b**)) extending into the right atrium (white star). A large left renal cyst is also noted (black star). T1 (**c**) and T2 (**d**) weighted image (WI) with fat saturation in the axial plane with arrows pointing to multiple retroperitoneal lymph node metastases; the left renal cyst is also depicted (black star). L = liver, Sp = spleen, St = stomach, rK = right kidney.

The unenhanced MRI examination described a mass at the upper pole of the left kidney with necrotic and hemorrhagic areas, likely exhibiting extracapsular extension. The tumor was associated with a voluminous cystic structure, without the possibility to exclude secondary invasion of the renal cyst based on imaging. Complete invasion of the left renal vein and supra-renal inferior vena cava was noted, extending up to the level of the right atrium. Additionally, there was evidence of retroperitoneal lymphadenopathy (Figure 5).

PET-CT examination was the investigation that definitively staged the renal tumor as T4N1M1, revealing a locally invasive renal mass, active metabolic involvement of supra- and sub-diaphragmatic lymph nodes, as well as an extensive intracardiac tumor thrombosis through the inferior vena cava.

Therefore, the initial staging of the left renal tumor, based on MRI and according to the TNM classification as T4N1Mx, has been subsequently re-staged after the PET-CT examination as T4N1M1.

The renal tumor is associated with a pro-coagulative state and the thrombus generates retrograde stasis. Moreover, the cancer cells associate adhesive interactions initiated by factors produced by both endothelium and cancer cells, including growth factors and a variety of cytokines [21]. Therefore, the case elements fit within Virchow’s triad.

### 1.4. Therapeutic Intervention

The presence of extensive IVC thrombosis observed during the ultrasound examination led to the initiation of anticoagulant treatment, which unfortunately resulted in a decrease in hemoglobin levels with approximately 2 g/dL, without externalization, both imaging investigations (CT and MRI) indicating signs of hemorrhage in the cystic mass. Therefore, the patient received a recommendation for low doses of LMWH at discharge, under hemoglobin level control.

Once the diagnosis was made clear, the patient was discharged from our hospital. He was then admitted to a multidisciplinary facility to evaluate the therapeutic options and for clinical follow-up. Following a thorough evaluation involving cardiovascular surgery, urology, nephrology, and oncology, the consensus was that the renal tumor had progressed beyond the possibility of curative therapeutic interventions. Surgical resection and thrombectomy were not considered feasible, given the disease stage and the fact that the risks outstand the benefits. In these conditions, a kinase inhibitor, Cabozantinib 60 mg/od, alongside low doses of LMWH represented the main therapy.

## 2. Discussion

### 2.1. Virchow–Troisier Nodules

Virchow–Troisier nodules are described as one or more nodules located above the median clavicular portion in the supraclavicular triangle or posterior to the clavicular bundle of the sternocleidomastoid muscle [22].

Isolated Virchow–Troisier adenopathy is mostly associated with infra-diaphragmatic neoplasia. The eponyms came from the two researchers who identified the pathological connections. The first description belongs to the pathologist Rudolf Ludwig Karl Virchow, who presented left supraclavicular adenopathy as an indicator of metastatic neoplasia, most commonly associated with gastric malignancy. Subsequently research, conducted by Charles-Emile Troisier, has shown that this type of adenopathy was also associated with other neoplasms apart from gastro-intestinal cancers, such as lung adenocarcinoma, prostate cancer, lymphomas, or infectious pathology (TB, syphilis) [22,23].

Virchow’s node is the last lymph node of the thoracic duct. It receives lymphatic drainage from the left side of the head, neck, thorax, abdomen, pelvis, and bilateral lower extremities, ultimately draining into the jugular–subclavian venous junction through the thoracic duct [22,23]. The mechanism of the appearance of this left supraclavicular adenopathy in the context of malignancy is most likely due to tumor embolization from primary sites through the thoracic duct, ultimately involving Virchow’s node, where some cancer cells remain trapped, causing an increase in its volume. The enlargement of Virchow’s node is known as Troisier’s sign [22].

Considering this, left supraclavicular adenopathy represented a warning sign in the case presented, leading to further paraclinical investigations. Left supraclavicular adenopathy is a clinical indication of an already advanced-stage neoplasm, in most cases an infra-diaphragmatic tumor [8]. Virchow–Troisier adenopathy can also lead to complications on its own, with the most common being thoracic outlet syndrome, Horner’s syndrome, and unilateral phrenic neuropathy [22], complications that were not detected in our patient.

Abdominal ultrasound ruled out gastrointestinal tumor pathology, and the normal PSA level and normal appearance of the prostate excluded prostate neoplasia. Instead, the abdominal ultrasound revealed a giant, lower pole, left renal transonic mass, and a heterogeneous mass at the upper pole of the left kidney, described on MRI as a tumor with necrotic and hemorrhagic areas, with possible extracapsular extension. The ultrasound detection of IVC and right cavity thrombosis, as well as left renal vein thrombosis (MRI), raised several issues of differential diagnosis until the result of the histopathological examination arrived.

### 2.2. Inferior Vena Cava Thrombosis

IVC thrombosis is highly associated with neoplastic disease. But despite this fact, IVC thrombosis is identified in only 0.07% of hospitalized patients with cancers. Notably, IVC thrombosis related to malignancy more often extends into the right atrium [24]. The most frequent malignancies related to IVC thrombosis are represented by RCCs (38%), genitourinary cancers (25%) [25], bronchus and lung cancers [26], retroperitoneal leiomyosarcoma [27], and adrenal cortical carcinoma [28].

Correlating the information gathered from the clinical examination, ultrasound, and imaging and analyzing the appearance and location of the retroperitoneal tumor mass, suspicion of soft tissue sarcoma was raised. Soft tissue sarcoma with retroperitoneal localization is rare (constituting approximately 1% of all solid tumors), with an incidence of 2 cases/100,000 people [29] and an almost equal distribution between genders, with the majority of patients aged between 54 and 65 years [30]. Approximately 70% of soft tissue tumors with retroperitoneal localization are malignant, with the most common histological types being leiomyosarcoma and liposarcoma [31]. The massive invasion into the IVC has led to considering leiomyosarcoma as the primary diagnosis.

Retroperitoneal leiomyosarcomas often originate in the inferior vena cava, its tributaries, or smaller vessels [32]. They are commonly an incidental finding in imaging [30]. When symptomatic, they frequently exert a mass effect, and clinically, abdominal pain, nausea, vomiting, anorexia, weight loss, fatigue, lower extremity edema, and other signs of stasis can occur, with diagnosis typically made when they reach a significant size [32]. CT and MRI are the first-line imaging methods [33]. They are performed in order to detect the local extent and distant metastases of the tumor and for preoperative surgical planning [33,34].

First-line treatment consists of surgical resection, aiming for negative margins. Radical surgery is often needed, including the resection of the involved organs. For tumors with vascular involvement or of vascular origin, surgery involves resection of the tumor with either vessel reconstruction or ligation. Chemotherapy or radiation therapy can be used in neoadjuvant or adjuvant settings [30].

Regarding the differential diagnosis, in our case, lymphoma or metastatic testicular cancer (presenting as a retroperitoneal tumor mass) should be ruled out [35]. These, and other causes such as duodenal tumors and pancreatic tumors, were excluded by clinical presentation, imaging, and histopathology and immunohistochemical analysis.

Furthermore, IVC thrombosis can be interpreted in the context of extending thrombosis from the left renal vein (as described in the MRI examination), the neoplasm being the pathological determinant of the procoagulant state [36]. Virchow’s triad refers to the three main factors involved in thrombosis: venous stasis, endothelial injury, and hypercoagulability. In the context of a neoplasm, a procoagulant state itself, these factors can play an important role in the development of thrombosis and its associated complications [36].

In this case, both ultrasound and MRI revealed a highly suggestive aspect for renal vein and IVC thrombosis, which could be supported by the following argument: the tumor invasion of the left renal vein can lead to venous stasis, promoting thrombus formation by interfering with the velocity of the blood flow and accumulation of procoagulant factors in the affected area. Malignant tumors, such as the one described in this case, can cause injuries to the vascular endothelium. These injuries trigger an inflammatory response that can activate the coagulation cascade, favoring thrombosis. The tumor itself may secrete procoagulant substances that can damage the vascular endothelium [36].

Oncology patients often present a state of hypercoagulability determined by the production of procoagulant factors such as tissue factor, associated with an increased risk of thrombosis [37]. Neoplastic tumors can release procoagulant substances or induce a systemic inflammatory reaction, resulting in the activation of the coagulation cascade [36]. The reverse is also true: approximately 20% of patients with symptomatic venous thrombosis have an active neoplasm. Complications of thrombosis often occur concurrently with the cancer diagnosis, but in certain cases, they can precede the diagnosis. The implications of Virchow’s triad in neoplasms are significant because cancer-associated thrombosis can have serious consequences, one of them being pulmonary embolism, which can be fatal [36].

In the case of patients with suspected thrombosis, several paraclinical investigations can support the diagnosis: ultrasound—which in the presented case showed an image suggestive of extensive thrombosis from the left renal vein, through the inferior vena cava, up to the right atrium protruding into the right ventricle during diastole; D-dimers—with elevated values (6.6 µg/mL); and MRI or CT, with MRI having higher sensitivity in detecting venous thrombosis. Another diagnostic method is represented by angiographic exploration [36].

From an epidemiological perspective, renal neoplasms occur with a frequency two times higher among the male population, with an average onset age of around 64 years [38].

Among the known risk factors for the occurrence of renal neoplasms, we mention the following: smoking (associated with more advanced disease); hypertension; obesity—elevated BMI is associated with less advanced stage of renal neoplasms but with increased risk for neoplastic disease [39,40]; polycystic kidney disease and chronic kidney disease; occupational exposure to toxic compounds such as cadmium, asbestos, and petroleum; prolonged use of analgesics, especially compounds containing phenacetin and aspirin, which can lead to chronic kidney disease with an increased risk for urothelial and renal pelvis tumors; genetic factors (first-degree relatives with oncologic pathology, disease onset before the age of 40, and bilateral or multifocal disease); sickle cell disease; and kidney stones [38].

Imaging investigations (ultrasound, CT, and MRI) highlighted a malignant left renal tumor. In this context, it should be noted that renal neoplasms are classified into numerous histological types, each with different macroscopic and microscopic characteristics, evolution, and treatment.

### 2.3. Histological Subtypes of Renal Carcinomas

The histological classification of renal carcinomas is of particular importance, considering that the subtypes of renal cancer have different treatment methods and varying prognoses. Imaging also plays a significant role in the staging and follow-up of patients with renal neoplasms [41]. The main subtypes of renal carcinomas are [41] clear cell renal carcinoma (75%), papillary renal carcinoma (10%), chromophobe renal carcinoma (5%); cystic-solid renal carcinoma (1–4%), and Bellini duct renal carcinoma (1%). Renal carcinomas with MiT gene translocation and renal carcinoma associated with neuroblastoma are rare subtypes. Unclassified renal carcinomas represent 4–6% of cases [41].

Clear cell renal carcinoma mostly occurs sporadically while 5% of cases are associated with Von Hippel–Lindau syndrome or tuberous sclerosis, as well as other hereditary diseases. Macroscopically, it is a solid, yellowish tumor with areas of necrosis, hemorrhage, or cystic degeneration. Histologically, it is composed of clear cells, a feature attributed to the rich cytoplasmic content of lipids and glycogen. Imaging shows intense contrast enhancement in the corticomedullary phase and a wash-out phenomenon in the nephrogenic phase. It is characterized by tumor extension into perirenal fat tissue and invasion of the renal vein and inferior vena cava and is [41] immunohistochemically characterized by positive staining for CA-IX, CD10, and vimentin and negative for CK7 [41,42].

Papillary renal carcinoma is the second most common subtype of renal cancer. The type 1 (basophilic) class is characterized by a single row of basophilic cells arranged on the basement membrane, with clear cytoplasm and hyperchromatic nuclei, while type 2 (eosinophilic) is characterized by cells with granular eosinophilic cytoplasm, prominent nucleoli, and areas of necrosis. Type 1 is usually diagnosed at earlier stages, thus having a better prognosis compared to type 2. Papillary renal carcinoma is often a solid, well-defined tumor with slow growth. Imaging-wise, they appear more homogeneous compared to clear cell renal carcinomas and have poor vascularity [41]. Immunohistochemically, they are positive for CK7 and AMACR [42].

Chromophobe renal carcinoma has the best prognosis and is the least aggressive renal carcinoma. It originates from the intercalated cells of the collecting ducts and is characterized by large pale cells with reticulated cytoplasm and a perinuclear halo. It tends to be more homogeneous compared to clear cell carcinomas, with weaker contrast uptake than this type, but more prominent than papillary carcinomas [41]. It shows positive staining for c-KIT and CK7 [42].

Bellini duct renal carcinoma is a rare but highly aggressive subtype, with 30% of patients already having metastases at the time of diagnosis. It is characterized by an irregular cellular infiltrate in the walls of the collecting ducts with desmoplastic reaction. Imaging-wise, they appear as heterogeneous and hypovascular lesions [41]. The immunohistochemical phenotype is characterized by positive staining for high-molecular-weight cytokeratin and CK7 [42].

Renal cell carcinoma with Xp11.2 translocation with TFE3 gene **fusion** is a rare subtype of renal carcinoma that occurs more frequently in the pediatric population (20–25% of kidney cancer cases in children compared to 1–4% in adults), with a worse prognosis in adult patients compared to children. In the adult population, the incidence is higher in females, and the average age of onset is 30–40 years. The only recognized risk factor is exposure to cytotoxic chemotherapy in childhood. Histologically, it is characterized by well-defined papillary structures composed of epithelioid cells. The cells contain clear/eosinophilic cytoplasm, visible nucleoli, and psammoma bodies. Imaging is often non-specific [7,43]. The specific immunohistochemical marker for this type of renal carcinoma is positive staining for the TFE3 protein [42].

Considering that the subtypes of renal cell carcinomas presented above cannot be clearly differentiated through imaging studies or clinical characteristics, but only through histopathological and immunohistochemical analysis, these latter two investigations are essential for the diagnosis of a renal neoplasm.

### 2.4. Histopathological and Immunohistochemical Features

Renal carcinoma with Xp11.2 translocation involving TFE3 gene fusion was first recognized as a distinct entity in 2004, and later in 2016, it was classified in the category of renal carcinomas with MiT gene translocations, alongside renal carcinoma with transcription factor B (TFB) gene fusion. It is characterized by the fusion of the transcription factor E3 gene located on chromosome Xp11.2 with various partner genes. At least 6 such translocations have been identified and characterized according to the literature data (e.g., the PRCC gene, ASPL gene, and SFPQ gene). It is noteworthy that ASPL-TFE3 gene fusion is also implicated in the development of soft tissue sarcoma [6,7].

Clinically, the disease can progress indolently and have a very slow evolution or rapid progression, with the latter situation carrying a reserved prognosis. Patients may present with specific symptoms and signs described for renal tumors, such as lower back pain, hematuria, and a palpable painless abdominal mass, or they can be completely asymptomatic [7,43]. In the case mentioned above, the patient presented with fatigue, asthenia, and weight loss, without macroscopic or microscopic hematuria. However, these nonspecific symptoms indicated a possible neoplasm, and the identification of left supraclavicular lymphadenopathy supported this suspicion.

The imaging methods used for diagnosis are numerous and include ultrasound, CT, MRI, and PET-CT. These methods help determine the exact location of the tumor, the degree of invasion, and the presence of distant metastases. The optimal treatment method is subsequently decided based on TNM staging. As mentioned earlier, imaging cannot differentiate between the subtypes of renal carcinoma, thus requiring additional investigations [6,7,43].

Identifying histopathological and immunohistochemical characteristics is the most important step in diagnosing this type of renal carcinoma [42]. Macroscopically, the tumor appears yellow-brown, with areas of necrosis, hemorrhage, or cystic regions on its surface. Microscopically, it can easily mimic any type of renal cancer, cytologically being characterized by well-defined papillary structures composed of epithelioid cells. The cells contain clear/eosinophilic cytoplasm, visible nucleoli, and psammoma bodies. Architecturally, structures with solid, nest-like, trabecular, or cystic patterns have been described, and these structural variations are associated with different types of chromosomal translocations [7,43].

Immunohistochemical examination has long been the gold standard for diagnosing renal carcinoma with Xp11.2 translocation. However, due to numerous false-positive or false-negative results obtained from immunohistochemical examination, it was later replaced by fluorescent in situ hybridization (FISH) analysis, which detects the gene’s karyotype with higher accuracy. Immunohistochemically, this type of renal cancer exhibits the following characteristics: PAX+, Vimentin+, CK7–, CA IX–, CD10+, and TFE3+. Positive staining for the TFE3 protein is specific to renal carcinoma with Xp11.2 translocation and is not found in conventional renal carcinomas. Therefore, it was long considered the defining element of diagnosis [6].

The differential diagnosis of this type of renal tumor from conventional types of renal carcinomas is based on histopathological characteristics combined with immunohistochemical markers. Renal carcinoma with Xp11.2 translocation involving TFE3 gene fusion presents variable clinical manifestations compared to conventional renal cancers, most likely due to the heterogenicity of the tissue structure [43].

The most important immunohistochemical marker in differentiating renal carcinoma with Xp11.2 translocation from other types remains positive staining for the TFE3 protein [6,7,42,43].

If this is not identified, the differential diagnosis becomes more challenging, but previous studies have identified some specific immunohistochemical patterns for renal carcinoma with TFE3 gene fusion. In general, the expression of cytokeratins (AE1/AE3, Cam 5.2, CK7, EMA) and melanocytic markers (HMB-45 and Melan-A) is negative, while the expression of vimentin, CD10, and E-cadherin is positive. The absence of CK7 and EMA and the overexpression of E-cadherin and CD10 are helpful elements in differentiating renal carcinomas with Xp11.2 translocation from conventional clear cell renal carcinomas (CK7-, EMA+, E-cadherin-, CD10+) or papillary renal carcinomas (CK7+, E-cadherin+) [7].

Elevated vimentin expression has been associated with metastatic potential and poor prognosis in various cancer types, including liver, breast, lung, and prostate cancers. It was recently discovered that the loss of vimentin in preoperative biopsies serves as an independent predictor of poor prognosis and lymph node metastases [44].

As it was shown in an observational study of 91 patients that the presence of TFE 3 is associated with a faster rate of progression of RCC, with lymph node and distant metastasis occurring more often and more rapidly comparing with patients with TFE3-negative RCCs. Also, the study demonstrated that TFE3-positive expression was an independent prognostic factor associated with poor progression-free survival [45]. Large studies have shown that survival in patients with TFE3-rearranged RCC was similar to TGE-rearranged negative clear cell RCC but worse than in patients with TFE-rearranged negative papillary RCC [46].

Another study analyzed the prognostic value of Carbonic Anhydrase IX. The results showed that low CA IX expression was associated with poor disease-specific survival, unfavorable progression-free survival, and worse overall survival. Furthermore, low CAIX expression was significantly associated with the presence of lymph node and distant metastases and predicted a higher tumor grade [47]. CA IX expression has demonstrated a predictive role in improved survival and response to treatment with IL-2 [48]. Conversely, decreased expression was linked to poor survival rates in patients with advanced RCC [49].

The above-mentioned immunohistochemicals are crucial in the prognosis and treatment of TFE3-rearranged RCC. TFE3-rearranged RCC often presents in younger patients, and its behavior can range from indolent to aggressive, depending on the specific gene fusion. Vimentin highlights the potential invasiveness and metastatic capabilities of the tumor while CA IX suggests a role for metabolic adaptations that may influence therapy responses [50].

From a treatment perspective, these markers guide personalized strategies. While no specific FDA-approved therapies for TFE3-rearranged RCC are formulated, its molecular features suggest potential benefits from MET or VEGF pathway inhibitors, while immunotherapy with immune checkpoint inhibitors is also an alternative. Furthermore, the focal expression of CA IX makes anti-CA IX therapies a reliable option [51].

By identifying markers linked to tumor aggressiveness, such as TFE3 and Vimentin, clinicians can tailor monitoring and therapeutic interventions, optimizing outcomes for patients with this rare RCC subtype [52]. Immunohistochemical patterns should be correlated with histopathological features for an accurate diagnosis [7].

As of now, there is no known optimal treatment for RCCs with Xp11.2 translocation. The most effective treatment method is surgical removal of the tumor, feasible only when the tumor is localized without invasion into neighboring structures. Invasive tumors or non-invasive tumors, but with distant metastases, have shown positive outcomes with therapies involving tyrosine kinase inhibitors, vascular endothelial growth factor (VEGF) receptor inhibitors, and mTOR inhibitors [53,54,55]. There are cases in the literature that describe patients who received immunomodulatory treatment (checkpoint inhibitors) or mTOR inhibitors, but the benefits have not been consistently satisfactory [6,7,43]. Additionally, several studies have demonstrated that patients with renal cell carcinoma with Xp11.2 translocation with metastases that have been treated with Cabozantinib, a c-Met and VEGFR2 tyrosine kinase inhibitor, had better and more durable outcomes compared to those treated with other VEGF inhibitors or other tyrosine kinase inhibitors [56,57].

Future research in Xp11.2 translocation RCC should focus on depicting the molecular mechanisms and diversity of TFE3 gene fusions. Investigating how different fusion partners affect prognosis, tumor progression, and therapeutic responsiveness can provide a clearer understanding of this rare cancer subtype. Larger research efforts are needed to establish a comprehensive framework for understanding this rare RCC variant [58,59].

The presented clinical and paraclinical data fill a critical gap in the literature by providing insights into this rare and poorly understood malignancy. The detailed case analysis offers a foundation for future research and advocates for a concerted effort to study this cancer subtype more comprehensively, which could ultimately lead to innovations in both diagnostic and therapeutic approaches, improving patient outcomes.

Our case report contributes to the limited existing literature on Xp11.2 translocation RCC by presenting detailed histological, immunohistochemical, clinical, and imaging findings. The study reinforces the importance of using a multidisciplinary diagnostic approach, and the application of markers such as TFE3, PAX8, and CA IX helps confirm the diagnosis and also delineates it from other renal tumors. We have also highlighted the importance of accurate differentiation from other metastatic malignancies, which is vital for choosing the appropriate treatment strategy.

## 3. Conclusions

We presented a rare type of renal carcinoma, which was not identified through specific signs as hematuria but rather through the presence of a Virchow node and thrombosis of the inferior vena cava extending into the right heart chambers. In certain situations, excision and biopsy of the Virchow node may be the only available method for histopathological and immunohistochemical diagnosis. Inferior vena cava thrombosis can be the result of numerous malignant pathologies and may lead to life-threatening complications. Further studies should be conducted regarding the optimal treatment for patients with renal carcinoma associated with Xp11.2 translocation/gene fusion TFE3, considering that the literature data are lacking. Most importantly, a comprehensive physical examination and clinical judgment remain essential for achieving an accurate diagnosis, even in the age of advanced and sensitive imaging techniques.

## Figures and Tables

**Table 1:** Biological parameters.

Parameter	Value	Normal Value
RBC	9.20 g/dL	12.0–15.0 g/dL
MCV	84.60 fL	80–96 fL
MCH	27.30 pg	27–33 pg
PLT	254,000/mL	150–400 × 10^3^/µL
LYMF%	9%	20–45%
NEUT%	84.8%	43–65%
EO%	0.7%	1–5%
ALT	13.00 U/L	1–35 U/L
AST	18.00 U/L	14–36 U/L
Glycemia	90.9 mg/dL	65–105 mg/dL
Total cholesterol	121 mg/dL	0–200 mg/dL
PSA	1.09 ng/dL	0–4 ng/mL
Iron	25.30 µg/dL	37–170 µg/dL
HDL cholesterol	36 mg/dL	>60 mg/dL
LDL cholesterol	63 mg/dL	0–99 mg/dL
Urea	58.60 mg/dL	15–36 mg/dL
ESR	75 mm/h	6–11 mm/h
D-dimers	6.60 µg/mL	0–0.50 µg/mL
Fibrinogen	626.00 mg/dL	150–400 mg/dL
CRP	2.29 mg/dL	0–0.32 mg/dL
Creatinine	1.84 mg/dL	0.6–1.2 mg/dL
GFR	37.36 mL/min	75–115 mL/min
K	4.13 mmol/L	3.6–5 mmol/L
Na	141.70 mmol/L	137–145 mmol/L
Uric acid	7.74 mg/dL	2.5–6.2 mg/dL

**Table 2:** Complete urinalysis and urine culture.

Parameter	Value	Normal Value
SG	1.01	1.016–1.022
Ph	7.00	4.8–7.4
LEU	Negative	NEGATIVE
NIT	Negative	NEGATIVE
PRO	500.00	-NEGATIVE/mg/dL
GLU	Normal	-<30/mg/dL
KET	Negative	-NEGATIVE
UBG	Normal	=<1/mg/dL
BIL	Negative	-NEGATIVE
ERY	Negative	-NEGATIVE
Epithelia	Relatively frequent	
Leukocytes	Rare	
Salts	Rare amorphous urates	
Urine culture	<1000 CFU/mL. Screening for MRSA, Enterobacteriaceae, *Acinetobacter* spp., and *Pseudomonas* spp. is negative	

## Data Availability

The dataset will be provided by the corresponding authors on reasonable request.
